# Housing instability patterns among low-income, urban Black young adults in California and associations with mental health outcomes: baseline data from a randomized waitlist-controlled trial

**DOI:** 10.1186/s12889-024-19948-y

**Published:** 2024-09-13

**Authors:** Michelle K. Nakphong, D. Jovon Bright, Ala Koreitem, A. Rain Mocello, Nadra E. Lisha, Hannah H. Leslie, Itzel Estrada, Margaret K. Libby, Sheri A. Lippman, Marguerita A. Lightfoot

**Affiliations:** 1 Division of Prevention Science, Department of Medicine, University of California, San Francisco, San Francisco, CA USA; 2 MyPath, San Francisco, CA USA; 3 Oregon Health & Science University - Portland State University, Portland, USA

**Keywords:** Young adult, Housing, Mental health, Housing instability, Depression, Social discrimination, Latent class analysis, Anxiety, Racial disparities

## Abstract

**Background:**

Deep-rooted racial residential segregation and housing discrimination have given rise to housing disparities among low-income Black young adults in the US. Most studies have focused on single dimensions of housing instability, and thus provide a partial view of how Black young adults experience multiple, and perhaps overlapping, experiences of housing instability including homelessness, frequent moves, unaffordability, or evictions. We aimed to illuminate the multiple forms of housing instability that Black young adults contend with and examine relationships between housing instability and mental health outcomes.

**Methods:**

Using baseline data from the Black Economic Equity Movement (BEEM) guaranteed income trial with 300 urban low-income Black young adults (aged 18–24), we conducted a three-stage latent class analysis using nine housing instability indicators. We identified distinct patterns by using fit indices and theory to determine the optimal number of latent classes. We then used multinomial logistic regression to identify subpopulations disproportionately represented within unstable housing patterns. Finally, we estimated associations between housing experience patterns and mental health outcomes: depression, anxiety, and hope.

**Results:**

We found high prevalence of housing instability with 27.3% of participants reporting experiences of homelessness in the prior year and 39.0% of participants reporting multiple measures of housing instability. We found the 4-class solution to be the best fitting model for the data based on fit indices and theory. Latent classes were characterized as four housing experience patterns: 1) more stably housed, 2) unaffordable and overcrowded housing, 3) mainly unhoused, and 4) multiple dimensions of housing instability. Those experiencing unaffordable and overcrowded housing and being mainly unhoused were more than four times as likely to have symptoms of depression (Unaffordable: aOR = 4.57, 95% CI: 1.64, 12.72; Unhoused: aOR = 4.67, 95% CI:1.18, 18.48) and more than twice as likely to report anxiety (Unaffordable: aOR = 2.28, 95% CI: 1.03, 5.04; Unhoused: aOR = 3.36, 95% CI: 1.12, 10.05) compared to the more stably housed pattern. We found that hope scores were similarly high across patterns.

**Conclusions:**

High prevalence of housing instability and mental health challenges among low-income Black young adults demands tailored interventions to reduce instability, given widening racial disparities and implications for future well-being into adulthood.

**Supplementary Information:**

The online version contains supplementary material available at 10.1186/s12889-024-19948-y.

## Introduction

Black young adults disproportionately experience housing instability precipitated by a long history of structural racism in the US. Over one-third (35%) of youth (those under age 25) experiencing homelessness in the U.S. identify as Black despite comprising only 14% of the total youth population [1]. Once unhoused, Black youth are also 69% more likely to reenter homelessness than their White peers, highlighting their vulnerability to chronic instability [2]. Structural racism codified in housing policies, legacies of redlining, discriminatory lending practices, and devaluation of assets in predominantly Black neighborhoods has given rise to high levels of housing instability among Black young adults aged 18–24 [3–5]. Moreover, institutionalized racism *continues* to drive housing disparities such as lending institutions pushing Black borrowers into high-cost, subprime mortgages [6, 7].

Housing inequities drive and contribute to a host of racial disparities in outcomes for Black young adults. The racial homeownership gap is also widening; Black homeownership in the US dropped 4.8 percentage points between 2000–2017, now trailing White ownership by 30 percentage points [8]. Lack of homeownership is the primary driver of the widening racial wealth gap in which Black households have one-sixth the wealth of White households, contributing to intergenerational poverty and financial vulnerability [9]. Housing inequities also drive inequalities in Black young adults’ access to other resources, influencing their access to health care, quality education, decent employment, and political representation [10, 11]. Despite knowledge of deep-rooted racial housing disparities, literature and policy have focused narrowly on Black young adults experiencing homelessness [12–15]; the breadth of experiences of housing instability and their effects on health are under-researched and not well understood.

Most studies have focused on single dimensions of housing instability or specific subpopulations, such as unhoused populations, and we thus only have a partial view of how Black young adults experience multiple, and perhaps overlapping, experiences of housing instability including homelessness, frequent moves, living in overcrowded housing, or evictions.

New evidence sheds light on inequalities across forms of housing instability which are largely obscured by dichotomized classifications of housed or unhoused [16, 17]; over half (52%) of Black young adults report little or no confidence in their ability to pay next month’s rent—nearly three times the rate of Whites [18]. This nascent evidence reveals that Black young adults are highly vulnerable to multiple forms of housing instability which often precede (or follow) homelessness. Recent studies have also highlighted the need to conceptualize housing instability as a multidimensional construct, finding that experiences of instability are widespread, but vary greatly by type and severity [19, 20].

In addition, racial disparities in housing instability likely contribute to high levels of mental health difficulties among Black young adults during a critical period of establishing independence and health over the life course, but the linkages between multiple, overlapping experiences of housing instability and mental health disparities are under-researched [21–23]. This is particularly important given that the National Institute of Mental Health has prioritized advancing scientific knowledge that inform policies that will reduce youth mental health disparities in the US, especially among communities disproportionately affected by racism, discrimination, and other adverse experiences [24]. Notably, over two in five Black young adults report depressive symptoms (43%), and similar proportions (41%) report anxiety disorders [18], but receive treatment at much lower rates than their White counterparts when needed (31.7% vs. 45.1%) [25]. Black young adults also face persistent barriers to accessing mental health care and have elevated risk of developing chronic mental disorders in adulthood compared to other racial groups [26–28]. In contrast, understanding Black young adults’ levels of hope in relation to their housing can shed light on resilience [29]. Knowledge of Black young adults’ multidimensional patterns of housing instability and how they relate to mental health outcomes will advance our understanding of housing as a social determinant of health and provide essential information for designing effective tailored interventions that can reduce housing instability.

The primary objective of this study was to illuminate the multiple forms of housing instability that Black young adults contend with, identify distinct patterns of instability, and examine relationships between housing instability and mental health outcomes including symptoms of depression and anxiety as well as hope. This detailed examination of the housing patterns of Black young adults can be used to shape programs and policies that can prevent and alleviate housing instability, including more severe forms such as homelessness. In addition, exploring the relationship between housing instability patterns and mental health during a sensitive period of development—the transition to adulthood and independence—can shed light on how racial disparities in housing instability contribute to disparities in mental health over the life course.

## Methods

### Study setting

San Francisco and Oakland, CA both face sustained housing crises and displacement of communities of color; neither are able to keep pace with the growing levels of housing instability [17, 30]. Both cities have prioritized addressing housing affordability and homelessness, but have struggled to meet the housing needs of low-income residents. Despite political action and heavy financial investment, current strategies have been unable to tackle growing homelessness. For example, Oakland is in the midst of implementing an ambitious 5-year county plan involving ten key system programs including permanent supportive housing, crisis response beds, and transitional housing for youth [31], but the number of individuals experiencing homelessness has risen 9% between 2022 and 2024 [32, 33]. In San Francisco, the city has allocated upwards of $600 million annually to address homelessness and supportive housing, yet for every unhoused household that the city can place in housing, an additional four households become unhoused [17, 34]. In these cities, Black residents are also particularly vulnerable to the negative effects of increasing gentrification [35], and often left out from reaping the benefits of economic growth [36]. During the period of data collection, a COVID-19 emergency eviction moratorium that protected tenants from being evicted due to rent non-payment was in place beginning in March 2020 and extending to May 2023 in Oakland and until August 2023 in San Francisco.

### Data and population

We used baseline data from the Black Economic Equity Movement (BEEM) Project, a guaranteed income trial among low-income Black young adults aged 18–24 in San Francisco and Oakland, California [37]. In partnership with local youth-serving agencies, participants were recruited from Low-Income Housing Tax Credit (LIHTC) qualified census tracts, where 50% of households have incomes below 60% of the Area Median Gross Income (AMGI) or have a poverty rate of 25% of more [38]. Interested applicants completed a webform to assess initial eligibility and were then contacted by research staff by phone to complete eligibility screening.

Eligible applicants were 18–24 years old, identified as African American/Black, and had to be living in the US for at least 3 years without plans to leave the Bay Area. Applicants were required to provide documentation verifying their age and their home address or the address where they spend most nights, which was matched to eligible census tracts. If applicants were unhoused or unstably housed, a letter from a youth-serving agency endorsing their status as unhoused was required.

**Fig. 1:**
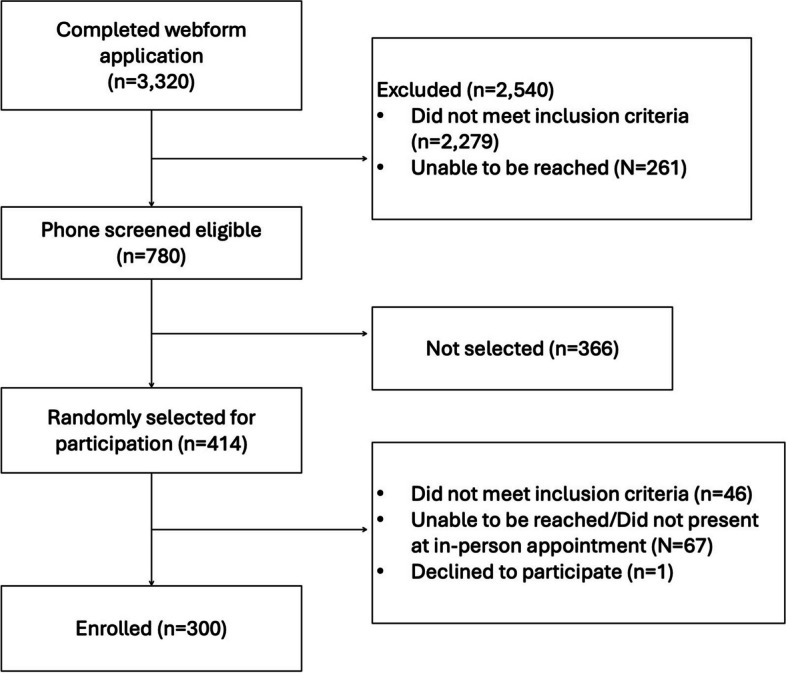
BEEM Project sampling process flowchart

Potentially eligible applicants were randomly selected from among the pool of eligible applicants balanced by gender, geographic location, and age, using REDCap (Research Electronic Data Capture) hosted at the University of California, San Francisco (UCSF) [39]. Selected participants were contacted via phone and scheduled for enrollment appointments at local sites (non-profits, universities) across the study area. Research assistants met participants in-person, verified eligibility using identification cards, residency, and/or agency letter, provided detailed information about the study, and obtained informed consent, resulting in a final sample of 300 participants. Details are provided in the sampling process flowchart (Fig. 1).

Participants completed baseline surveys via computer-aided personal interviewing (CAPI) using REDCap which lasted 45 min on average. Following survey completion, participants were randomized into intervention group (guaranteed income or waitlist control) and provided with resources for local services and agencies, as needed. Participants were compensated $40 for their time in cash or through CashApp (a mobile payment service that was recommended by young adults during study piloting) depending on participant preference. Enrollment took place between November 2022 and July 2023. Details on study design are published elsewhere [37].

### Community Working Group

This research was conducted in partnership with the Community Working Group (CWG), who provided input on the design, implementation, and interpretation of findings. The CWG comprises representatives from partnering youth-serving agencies, city officials in housing and human services, and young adults or near peers. The CWG provided input on the findings from this study, providing community perspectives on interpretation, framing of results, potential implications and directions for further investigation.

### Measures

#### Housing instability inputs

We drew from existing literature on housing instability and the Department of Housing and Urban Development (HUD) definitions and other housing instability studies to identify nine measures that capture multiple dimensions of housing instability (Table 1) [40–42]. *Currently unhoused* was measured using the HUD Category 1 definition of “Literally homeless” [40]. *Unhoused last year, frequent moves, moved due to cost*, *moved to share expenses, behind on rent last 3 months, currently behind on rent,* and *evicted* were measures drawn from literature on housing and health [41, 42]. *Overcrowded housing* was measured according to the HUD definition [43]. All input variables were constructed as binary variables and were coded ‘1’ if the participant reported an indictor and ‘0’ for if they did not. Eight measures (all but *currently unhoused*) were added to the baseline survey one month after study enrollment began, resulting in 16 participants missing data for those eight measures.

**Table 1:** Housing instability input measures

Indicator (*variable name*)	Item/Question	Response options and/or coding strategy
Currently unhoused (*unhoused*)	Respondents were asked: “Where do you live now? Pick the choice that best describes where you live.”	*Currently unhoused* were those who responded: shelter; transitional housing; hotel, motel, Airbnb, single-room occupancy (SRO) or youth hostel; street, squat, abandoned building; car *Currently housed* were those who responded: own apartment, room or house; parent’s home/apartment; someone else’s home/apartment; Group home
Unhoused in the last year (*unhoused last year*)	Have you experienced homelessness at any time in the last 12 months?	Yes/No
Lived in 3 or more places in the last 3 months (*frequent moves*)	In the last 3 months, how many places, including your current place, have you lived for one week or longer?	*Frequent moves* were those who responded 3 or more residences
Moved because of cost in the last 3 months (*moved due to cost*)	In the last 3 months, did you move because you could no longer afford the place (or places) where you were staying?	Yes/No
Moved to share expenses in the last 3 months (*moved to share expenses*)	Have you moved in with anyone in the last 3 months to share household expenses?	Yes/No
Gotten behind on rent in the last 3 months (*behind on rent last 3 months*)	In the last 3 months, have you ever gotten behind on your rent?	Yes/No
Currently behind on rent (*currently behind on rent*)	Are you currently behind on your rent?	Yes/No
Evicted in the last 3 months (*evicted*)	Have you been evicted at any time in the last 3 months? This could include you, or you and people you are living with. For example, if you lived in your parents’ home and they were evicted	Yes/No
Currently live in overcrowded housing (*overcrowded housing*)	How many people do you live with? In the place where you live, how many rooms are there? (including bedrooms, living rooms, etc.)	Those living in overcrowded housing were those who reported more individuals than rooms (i.e., > 1 person per room)

#### Mental health outcomes

We assessed depression using the 10-item Center for Epidemiologic Studies Depression scale revised for adolescents (CESDR-10), to which we added an item (“I felt alone in my life”) based on feedback from focus groups conducted prior to study recruitment. Respondents indicated on a scale of 0 to 3 how frequently in the previous week they had experienced each symptom (e.g., sadness, sleep difficulties, loss of interest, suicidality, etc.): rarely or none of the time (less than one day); some or a little of the time (1–2 days); occasionally or a moderate amount of time (3–4 days); or most or all of the time (5–7 days). We performed factor analysis to validate the performance of the scale in our study population and to check the fit of the additional item (CFI = 0.978, SRMR = 0.04, RMSEA = 0.074). Major, probable major, and possible major depressive episodes were defined as endorsing either anhedonia, dysphoria, or irritability nearly every day, plus the presence nearly every day of a total of: five or more symptoms (major), four symptoms (probable), or three symptoms (possible) [44]. For participants not captured in the preceding algorithm, we calculated the sum of all items to determine subthreshold (

$\ge$ 8 points) or no depression (< 8 points). We dichotomized the final variable into major and probable major vs. possible major depression, subthreshold or no depression.

We measured anxiety with the Generalized Anxiety Disorder scale (GAD-7) [45], which asks how often a participant had experienced a set of seven anxiety symptoms (e.g., feeling nervous, worrying, trouble relaxing, irritability, etc.) in the previous two weeks: not at all (0), several days (1), more than half the days (2), nearly every day (3). Scores were summed and dichotomized as no to mild anxiety (score $\le$ 9) and moderate to severe anxiety (score $\ge$ 10), based on published guidance by the scale authors [45].

Hope was measured by the Hope Matters Scale comprising 12 items capturing anticipation of a positive future, personal motivation to achieve goals, and the influence of others on hope [46]. A hope score was calculated from the 12 final items ranging from ‘1′ for total disagreement to ‘4′ for total agreement with all the hope items. We performed factor analysis to validate the performance of the scale in our study population (CFI = 0.981, SRMR = 0.048, RMSEA = 0.109).

#### Other variables

We also examined gender (man, woman, non-binary/transgender/other), age (18–20 years old vs. 21–24 years old), educational attainment (some post-secondary education or higher vs. high school or less), employment (full-time vs. part-time/miscellaneous income/unemployed), and living situations including living with parents (vs. not), and living with their own children (vs. not).

### Analyses

We performed univariate descriptive analyses to characterize the sample and assess variable distributions. We then performed a 3-step analysis to examine patterns of housing instability using Latent Class Analysis (LCA), a person-centered method which identifies individuals who can be grouped together based on common patterns of inputs, in this case were housing experiences [47]. Since LCA creates latent classes based on multiple indicators [48], it is particularly well suited to assess housing instability, a multidimensional construct. In the first stage, we used LCA to identify the number of distinct housing patterns (i.e., latent classes) present in the data by selecting the model that optimized model fit statistics according to Akaike Information Criterion (AIC), Bayesian Information Criterion (BIC), and entropy [49]. We examined a series of models beginning from a 2-class to a 5-class solution based on fit statistics indicating no further improvement in fit. We conducted the parametric bootstrap likelihood ratio difference test (BLRT) to compare the model with K classes (i.e., the class solution with the lowest AIC/BIC) to the models with K-1 classes and K + 1 classes, using 500 draws, 500 initial random starts, and 50 final stage optimizations [50]. We also examined whether missing data could be assumed to be Missing Completely At Random (MCAR) using Pearson Chi-square and Likelihood Ratio Tests (LRT); these tests indicated that data could be considered MCAR [51].

In the second stage, we used multinomial logistic regression to examine predictors associated with housing experience patterns to identify subpopulations at greater risk of experiencing unstable housing patterns. We used the automatic 3-step approach (R3STEP) in Mplus which has demonstrated good performance regarding bias, mean squared error, and confidence interval estimation [52]. For regression analyses, we designated the most stably housed pattern as the reference group based both on theory and because it was the modal class.

In the third stage, we estimated associations between housing experience patterns and mental health outcomes using the manual Bolck-Croon-Hagenaars (BCH) method [53]. The BCH method applies weights to account for the measurement error of the latent class variables and outperforms other methods because it more accurately determines the correct number of latent classes, especially in situations with sparse data or complex latent class models including those with covariates and outcome variables [49]. We performed the BCH method via a two-step weighted multiple group analysis: 1) estimating the LCA model and BCH weights, then 2) regressing the outcome variables on latent classes and covariates. Models for the binary depression and anxiety outcome variables employed a logit link. For the continuous hope outcome, we assessed means across latent classes using non-parametric Wald tests to compare differences across classes and levels. For each model, we excluded observations that were missing outcome data. We also included variables that were found to be significantly associated with latent classes in stage 2 as covariates in stage 3. Sensitivity analyses included fitting linear regression and multinomial logistic models with various constructions of mental health outcome variables (continuous score, categorical outcome). Results were consistent across models. Mplus (version 8.10) was used for all analyses [51].

## Results

### Sample characteristics

Among the 300 participants, 55.3% were 21–24 years old and 44.7% were 18–20 years old at enrollment (Table 2). 50.7% identified as women, 48.7% of participants identified as men, and 2.0% identified as non-binary, transgender or something else. Over half of participants (57.0%) reported living with their parents, 9.7% lived by themselves, 6.3% lived with their children and 4.7% lived with a partner. Most participants (63%) reported at least one housing instability measure and 39% of participants reported multiple measures. Participants most frequently reported living in overcrowded housing (30.0%), being behind on rent in the past three months (29.7%), and being unhoused in the past three months (27.3%). Participants least frequently reported being currently unhoused (8.7%) and getting evicted (2.0%). Participants also commonly reported negative mental health outcomes: 12.3% reported having major or probable major depression and 41.7% reported experiencing moderate or severe anxiety. On a scale of 1 to 4, the mean score for the hope measure was 3.35, indicating that, on average participants agreed with statements indicative of feeling hopeful about the future.

**Table 2:** Sample characteristics (*N* = 300)

Demographic Characteristics	N (%)
Gender	
Men	146 (48. 7%)
Women	148 (50. 7%)
Non-binary/transgender/other	6 (2.0%)
Age	
18–20	134 (44.7%)
21–24	166 (55.3%)
Educational attainment	
Did not complete high school or GED	16 (5.3%)
Completed high school or GED	167 (55.7%)
Some post-secondary education	117 (39.0%)
City of residence	
Oakland	196 (65.3%)
San Francisco	104 (34.7%)
Employment	
Employed full-time (FT)	39 (13.0%)
Employed less than FT	
Employed part-time	53 (17.7%)
Not employed	82 (27.3%)
Has income from miscellaneous jobs	113 (37.7%)
Missing	13 (4.3%)
Living arrangements^a^	
Lives by themselves	29 (9.7%)
Lives with parents	171 (57.0%)
Lives with partner	14 (4.7%)
Lives with own children	19 (6.3%)
**Housing instability inputs**	
Currently unhoused	26 (8.7%)
Unhoused in the past 12 months^b^	82 (27.3%)
Frequent moves^b^	25 (8.3%)
Moved because of cost ^b^	30 (10.0%)
Moved to share expenses^b^	32 (10.7%)
Behind on rent in 3 months^b^	89 (29.7%)
Currently behind on rent^b^	44 (14.7%)
Evicted^b^	6 (2.0%)
Live in overcrowded housing^b^	90 (30.0%)
**Mental Health outcomes**	
**Depression**	
No depression	93 (31.0%)
Sub-threshold or possible major depression	160 (53.3%)
Major or probable major	40 (13.3%)
Missing	7 (2.3%)
**Anxiety**	
No anxiety	75 (25.0%%)
Mild anxiety	91 (30.3%)
Moderate or severe anxiety	128 (42.7%)
Missing	6 (2.0%)
**Hope**	
Continuous score	Mean = 3.35, SD = 0.52
Missing	3 (1.0%)

^a^Not all types of arrangements are listed (e.g., roommates, extended family, etc.) and participants could check more than one option

^b^Item asked of *n* = 284

### LCA Findings

A four-class solution fit the data best according to AIC and BIC statistics (Supplement 1). Compared to the 3-class solution, the 4-class solution had a significantly lower AIC statistics, and BIC statistics were not significantly different (Supplement 1). The entropy value for the 4-class solution also showed that 89.4% of the sample could be accurately categorized on the basis of their class membership. Examination of the posterior probabilities also showed that participants had high probabilities of belonging to a single class, with coefficients ranging from 0.91 to 0.99. The parametric bootstrap likelihood ratio difference tests (4-class solution vs. 3-class & 4-class vs. 5-class) also indicated that the 4-class solution was better than the 3-class solution (*p* < 0.001) but that the 5-class solution was not better than the 4-class solution (*p* = 0.560). In addition, we considered the qualitative differences between classes within the 4-class solutions to be distinct, important, and meriting examination. For these theoretical and statistical reasons, we considered the 4-class solution to be the best fitting model for the data.

**Fig. 2:**
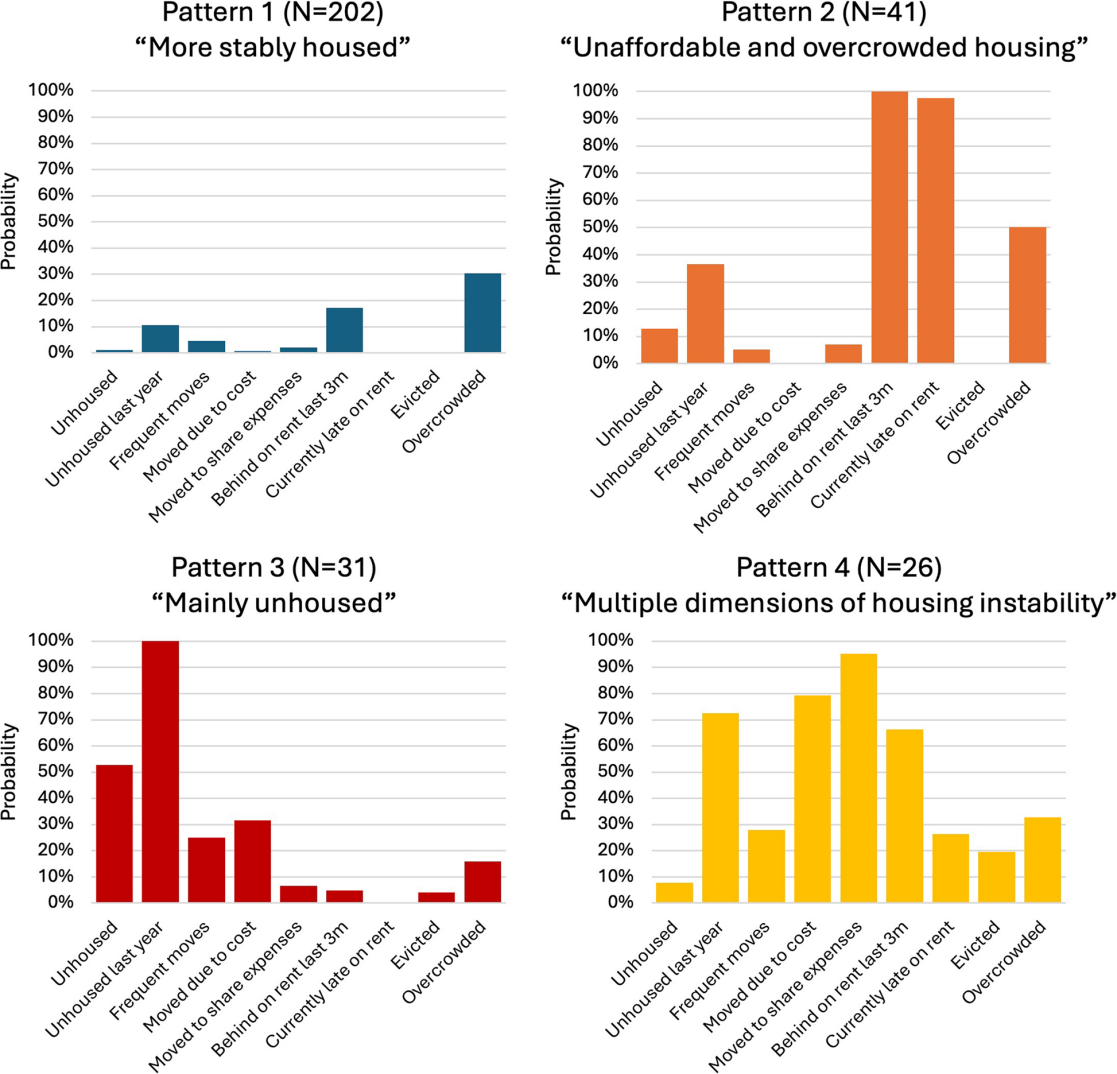
Housing experience patterns and estimated probabilities of reporting housing experiences: results of the LCA 4-class solution

Profiles of each of the four classes, which we refer to as patterns, are presented in Fig. 2, including each pattern’s probabilities of reporting housing experience inputs. We labelled Pattern 1 (67% of the sample) “More stably housed” since participants in this class had low probabilities across all housing inputs. Within Pattern 1, the input with the highest probability (30%) was living in overcrowded housing, which was still lower compared to other patterns. Pattern 2 (14%) “Unaffordable and overcrowded housing” participants had high probabilities of reporting being behind on rent in the last 3 months (100%), currently behind on rent (98%), and living in overcrowded housing (50%). Pattern 3 (10%) “Mainly unhoused” participants had high probabilities of reporting being currently unhoused (53%) and being unhoused in the last 12 months (100%). Pattern 4 (9%) “Multiple dimensions of housing instability” participants had high probabilities across being unhoused in the past 12 months (73%), moving because of cost (79%), moving to share expenses (95%), and being behind on rent in the last 3 months (66%).

### Associations between housing experience patterns and demographic characteristics

Housing patterns were associated with gender, education, age, and household characteristics in multivariable multinomial regression models (Table 3). Women had more than twice the odds of men (aOR = 2.56, 95% CI: 1.11, 5.86) to experience unaffordable and overcrowded housing compared to the more stably housed pattern. Women were also had more than twice the odds as men (aOR = 2.73, 95% CI: 0.99, 7.54) to experience multiple dimensions of housing instability compared to the more stably housed pattern, but this result was only marginally significant. Older individuals (aged 21–24) had more than three times the odds than younger (aged 18–20) (aOR = 3.66, 95% CI: 1.48, 9.06) to experience unaffordable and overcrowded housing and more than ten times the odds than younger (aOR = 10.81, 95% CI: 1.54, 75.81) to experience the main unhoused pattern, compared to the more stably housed pattern. Individuals who lived with their parents were also less likely to experience unaffordable and overcrowded housing (aOR = 0.31, 95% CI: 0.14, 0.69) and multiple dimensions of housing instability (aOR = 0.32, 95% CI: 0.12, 0.88) compared to the more stably housed pattern.

**Table 3:** Associations between demographic characteristics and housing experience groups, using Pattern 1 (More stably housed) as the reference

	Pattern 2 (Unaffordable and overcrowded housing)	Pattern 3 (Mainly unhoused)	Pattern 4 (Multiple dimensions of housing instability)
	**aOR (95%CI)**	**aOR (95%CI)**	**aOR (95%CI)**
Women (vs. men)	2.56* (1.11, 5.86)	1.44 (0.42, 4.97)	2.73 (0.99, 7.54)
Non-binary/non-conforming/etc. (vs. men)	4.11 (0.41, 5.86)	3.82 (0.12, 127.16)	—
Some postsecondary education (vs. high school diploma or less)	0.35* (0.14, 0.91)	0.20* (0.05, 0.81)	0.88 (0.32, 2.46)
Aged 21-24yo (vs. 18-20yo)	3.66** (1.48, 9.06)	10.81* (1.54, 75.81)	1.04 (0.38, 2.88)
San Francisco (vs. Oakland)	0.66 (0.28, 1.58)	3.18 (0.87, 11.63)	0.33 (0.09, 1.16)
Employed full-time (vs. part-time/not employed)	0.62 (0.20, 1.92)	0.69 (0.09, 5.55)	0.35 (0.05, 2.45)
Living with parents	0.31** (0.14, 0.69)	—	0.32* (0.12, 0.88)
Living with their own children	1.06 (0.24, 4.72)	3.99 (0.65, 24.37)	—

^*^p < 0.05, **p < 0.01, ***p < 0.001

### Associations between housing experience patterns and mental health outcomes

Multivariable regression analyses revealed that unstable housing patterns were associated with greater mental health challenges after adjusting for covariates (Table 4). Individuals experiencing unaffordable and overcrowded housing were more than four times as likely than the more stably housed pattern to report depressive symptoms (aOR = 4.57, 95% CI: 1.64, 12.72) more than twice as likely as the more stably housed pattern to report anxiety (aOR = 2.28, 95% CI: 1.03, 5.04). Those experiencing the mainly unhoused pattern were also more than four times as likely to report depressive symptoms (aOR = 4.67, 95% CI: 1.18, 18.48) and more than three times as likely to report anxiety (aOR = 3.36, 95% CI: 1.12, 10.05) compared to the more stably housed pattern. Those experiencing the multiple dimensions of housing instability pattern displayed elevated odds of depressive symptoms (aOR = 2.97, 95% CI: 0.91, 9.68) and anxiety compared to the more stably housed group (aOR = 1.20, 95% CI: 0.47, 3.06) but these estimates did reach not statistical significance.

**Table 4:** Associations between housing experience patterns and mental health outcomes

	**Housing patterns**		
	Pattern 2 (Unaffordable and overcrowded housing) vs. Pattern 1 (more stably housed)	Pattern 3 (Mainly unhoused) vs. Pattern 1 (more stably housed)	Pattern 4 (Multiple dimensions of housing instability) vs. Pattern 1 (more stably housed)
	**aOR (95%CI)**	**aOR (95%CI)**	**aOR (95%CI)**
**Depression (major/probably major vs. moderate/no depression)**	4.57 (1.64, 12.72)	4.67 (1.18, 18.48)	2.97 (0.91, 9.68)
**Anxiety (moderate/severe vs. no/mild anxiety)**	2.28 (1.03, 5.04)	3.36 (1.12, 10.05)	1.20 (0.47, 3.06)

All models adjusted for gender, education, age, and living with parents

Multivariable linear regression analyses with the hope score demonstrated that on average, participants across housing experience patterns agreed that they felt hopeful about the future. There were no significant differences in hope scores across housing experience patterns (Supplement 2).

## Discussion

Our study examined multiple dimensions of housing instability among a sample of urban, low-income Black young adults in northern California and identified distinct housing experience patterns and their associations with mental health. This is one of the first studies to measure housing instability in a population-based sample of low-income Black young adults who were both housed and unhoused, providing profiles of housing vulnerability and allowing us to compare factors across patterns [54]. Our study reveals unrecognized and underestimated prevalence of housing instability among low-income Black young adults over a relatively short period of time. Most (63%) participants reported at least one measure of housing instability and 39% of participants reported multiple measures over only 3 to 12 months. It is also critical to acknowledge the severity of housing instability within this population: over one-in-four participants (27%) reported being unhoused within the previous year. By comparison, it is estimated nationally that 1-in-10 young adults experience homelessness within a 12-month period [55].

Findings also emphasize the hidden experiences of housing instability among Black young adults. For example, San Francisco’s point-in-time counts find that less than 1% of residents are homeless at any given point [17]. By contrast, our study highlights the extent to which a large proportion of a sample of Black young adults cycles through episodes of homelessness. There was a high probability of being unhoused in the past year in all three of the unstable housing patterns and the study’s CWG noted that even those experiencing the more stably housed pattern had a 11% probability of being unhoused in the past year—challenging the notion that participants in this group were universally stably housed. Moreover, this study highlights the housing challenges that may precede or follow homelessness: particularly the unaffordability of rent/housing costs, mobility associated with high housing costs, and living in overcrowded housing.

Our results about disproportionate housing instability among women, older young adults (aged 21–24) and lower likelihood of instability among young adults living with a parent support other literature on unstably housed young adults. Black women face both a race and gender wage gap, [56] reflected by their overrepresentation in the unaffordable and overcrowded housing pattern. Findings may also illuminate housing discrimination against Black women, who are at greater risk of being evicted and denied rental applications than other racial and gender groups [57, 58]. The finding that older young adults are more likely to experience housing instability sheds light on the present difficulty of leaving family homes and transitioning to independence in a setting with few low-cost options and the vulnerability of those aging out of youth services, which also has implications for chronic housing instability [54]. However, those who live with parents are less likely to experience instability, emphasizing the importance of supportive parental relationships; parents can be key for youth and young adults exiting homelessness and transitioning to independent living [54, 59]. Conversely, lack of parental support can increase youth and young adults’ vulnerability to both adversity and housing instability [60, 61].

The high likelihood of mental health difficulties among low-income Black young adults experiencing unstable housing patterns highlights the vulnerability within this population. We found over four times the likelihood of depression and two to three times the likelihood of anxiety among those experiencing unaffordable and overcrowded housing and the mainly unhoused patterns compared to the more stably housed. We also found elevated depression in those experiencing multiple dimensions of housing instability although estimates did not reach statistical significance, likely because of the small size of the group. Studies assessing singular forms of housing instability have found associations with poorer mental health outcomes among young adults. For example, frequent moving during youth is associated with depression in young adulthood, with the likelihood of depression increasing 10% with each additional move [21]. Young adults who experience evictions are more than twice as likely to report concurrent depression and anxiety even years after an eviction compared to those who have not [62], and doubling up with extended family or non-kin increases risk of depression and poor health [63]. Our study extends this research by demonstrating that low-income Black young adults contend with high levels of depression and anxiety across distinct, multistoried patterns of housing instability.

Longitudinal and qualitative research is needed to elucidate how these distinct patterns and mental health difficulties contribute to one another. Lacking stable housing can expose youth to harm, inflict trauma, and contribute to worsening mental health, [64] but it is unclear whether similar or different exposures give rise to depression and anxiety across those different patterns of housing instability. In addition, qualitative inquiry may also provide insights on the specific challenges that Black young adults face. For example, people of color bear a double burden of stigma and discrimination from race and housing instability that contribute to greater psychological distress and poorer general health [65]. Findings from this study along with qualitative insights could be used for designing appropriate and accessible interventions that can address the distinct forms and patterns of housing instability and ultimately address mental health as well.

Notably, we also found high levels of hope across housing patterns despite differences in mental health symptoms. Among populations experiencing homelessness, hope has been studied as a critical component of goal-setting, future orientation, and resilience [66–68], which in turn influences positive emotional well-being and health behaviors [69, 70]. Hope can also rise and fall relative to youths’ social contexts, such as in response to major life events, social support, achieving success, or creative expression [71–73]. Nevertheless, there is mixed evidence on the relationship between hope and housing outcomes, potentially because of contextual differences. Some studies among adults experiencing homelessness have found that hope was associated with attaining housing goals [66], but others have found that hope and future orientation are unable to predict obtaining stable housing, especially when housing is scarce [68]. Particularly in this study, it is plausible that participants had higher levels of hope as they anticipated receiving guaranteed income within the parent BEEM project. Nascent evidence demonstrates positive impacts of guaranteed income on establishing housing stability supporting recipients’ ability to secure permanent housing, move into stable housing faster, and avoid eviction and homelessness [74–77]. Future research should further examine the impact of guaranteed income on sustained housing instability and the interplay with hope and the social context over time.

This study has some limitations related to measures, analysis, and the cross-sectional design. First, housing measures may still not be fully inclusive of the experiences of housing instability. For example, individuals tend to emphasize their own choice in moving and may not frame forced moves as evictions [78]. Individuals may also have different interpretations of ‘homelessness’ and thus may not consider certain arrangements, such as couch surfing or staying in a motel, as being unhoused [12, 79]. Our use of the GAD-7 for anxiety may also underestimate anxiety, especially since validation studies have found that Black respondents scored lower than those of other racial identities with similar general anxiety disorder symptoms [80]. However, we chose this measure because of its brevity and previous validation in similar populations, and we did not intend to make comparisons to other racial groups. Regarding analysis, our use of the data-driven LCA to identify patterns equally weighted each housing input measure, meaning that we were unable to account for varying severity of forms of housing instability such as being unhoused or living in overcrowded housing. In addition, the cross-sectional design of this study does not allow for causal conclusions about housing instability and mental health. Literature characterizes a bidirectional relationship such that housing instability and poorer mental health exacerbate one another [81, 82]. Future longitudinal research should test this hypothesis by investigating how the housing trajectories of participants in each of these patterns are associated with mental health outcomes over time. This study also took place during the COVID-19 pandemic in which many vulnerable populations experienced economic instability and poor mental health [83]. Future research should investigate whether the associations between unstable housing patterns and mental health difficulties are generalizable beyond this time period. Despite these limitations, this study illuminated hidden forms of housing instability and their relationships to mental health in a sample of urban, low-income Black young adults. Furthermore, sampling participants from two cities likely increases the generalizability of results to other urban metropolises in the US facing housing crises.

## Conclusions

This study underscores the lasting effects of racial discrimination manifested in the housing vulnerability of low-income Black young adults in the San Francisco Bay Area. Housing instability and high prevalence of mental health difficulties among low-income Black young adults demands urgent attention, especially given the stark and widening racial disparities and the implications for future well-being in adulthood. Experiencing housing instability during young adulthood amplifies risk of chronic housing instability and poor mental and physical health throughout adulthood [21, 30, 84, 85]. Housing assistance and mental health services should be complementary and paired with permanent supportive housing models, however, these services often prioritize those with severe mental illness or disabilities [86]. Given the concurrent housing and mental health needs of many low-income Black young adults, supportive housing interventions should broaden assistance to those struggling with housing instability across forms, potentially preventing severe types of instability, such as homelessness and severe mental illness [87]. Future research may explore how such programs can be designed to meet the needs of low-income Black young adults who experience unaffordable and overcrowded housing and multiple dimensions of instability (food insecurity and insufficient employment) in addition to those who are mainly unhoused.

Lastly, housing policy and development must better engage with young adults’ needs and trajectories as housing instability among young adults grows globally [88, 89] and homeownership is increasingly out of reach for young adults [90]. It is imperative that housing development and policy address the history of racial discrimination that has resulted in greater housing instability among Black communities. for instance, by increasing housing program participants’ choice of neighborhoods, inclusionary zoning, and addressing bias in financing [91]. Thoughtful, conscientious, and participatory action is needed to ensure a fair and stable future for low-income Black young adults.

## Supplementary Information

Supplementary Material 1.

Supplementary Material 2.

## Data Availability

The data are available from the corresponding author upon reasonable request.
