# Supplementary material for: Housing instability patterns among low-income, urban Black young adults in California and associations with mental health outcomes: baseline data from a randomized waitlist-controlled trial
Source: BMC Public Health. 2024 Sep 13;24:2492. doi: 10.1186/s12889-024-19948-y (PMC11396585; doi:10.1186/s12889-024-19948-y)
Supplement: Supplementary file 1 — Supplementary Material 1. [file 12889_2024_19948_MOESM1_ESM.pdf]

Supplement 1. Latent Class Analysis of housing patterns fit statistics

Model fit indices for latent class analysis of 2 to 5 solutions

Solution	AIC	BIC	Entropy	Log likelihood	BLRT p-value[1]
2-class	1968.649	2039.021	0.753	-965.3	–
3-class	1879.646	1987.056	0.885	-910.8	<0.001
4-class	1845.016	1989.463	0.894	-883.5	<0.001
5-class	1842.74	2024.226	0.917	-872.4	0.056

[1] p-value for the parametric bootstrap likelihood ratio difference test (BLRT) comparing the K class to the K-1 class.

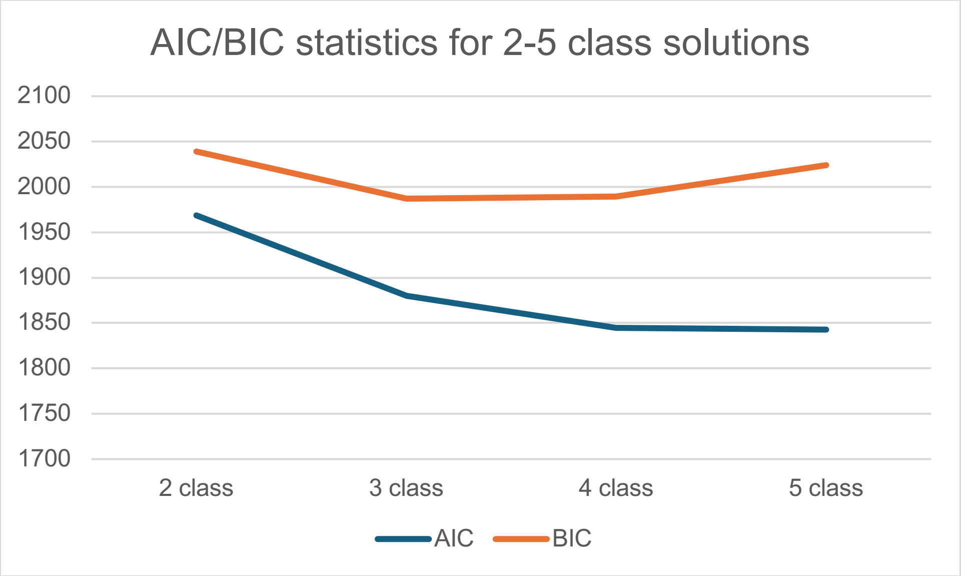

Average Latent Class Probabilities for Most Likely Latent Class Membership (Row) by Latent Class (Column) for the 4-class solution

	**Latent class**			
**Most likely latent class membership**	**1**	**2**	**3**	**4**
**1**	**0.946**	0.013	0.032	0.009
**2**	0.000	**0.989**	0.000	0.011
**3**	0.053	0.008	**0.911**	0.028
**4**	0.015	0.003	0.037	**0.945**
