# Supplementary material for: Housing instability patterns among low-income, urban Black young adults in California and associations with mental health outcomes: baseline data from a randomized waitlist-controlled trial
Source: BMC Public Health. 2024 Sep 13;24:2492. doi: 10.1186/s12889-024-19948-y (PMC11396585; doi:10.1186/s12889-024-19948-y)
Supplement: Supplementary file 2 — Supplementary Material 2. [file 12889_2024_19948_MOESM2_ESM.docx]

**Supplement 2.** Comparisons of mean hope scores across housing patterns

|  | **Hope (continuous)** | |
| --- | --- | --- |
| **Housing experience subgroups** | **Mean hope score**  **(95% CI)** | **p-value testing equivalence with Pattern 1 coefficient** |
| Pattern 1 (More stably housed) | 3.34  (3.27, 3.42) | — |
| Pattern 2 (Housing unaffordability and overcrowded) | 3.35  (3.17, 3.52) | 0.939 |
| Pattern 3 (Mainly unhoused) | 3.41  (3.18, 3.65) | 0.578 |
| Pattern 4 (Multiple dimensions of housing instability) | 3.30  (3.08, 3.52) | 0.758 |

Note: All models adjusted for gender, education, age, and living with parents.
